# Metal Ions in Longgu (Fossilized Bone) as a Natural Synergistic Network for Multi‐Target Therapy in Psychiatric Disorders

**DOI:** 10.1002/cbdv.71623

**Published:** 2026-09-04

**Authors:** Sun Xiujia, Li Zhan Hua, Gao Yuanze, Liu Jing, Li Li, Zhang Chao, Kumar Ganesan, Jianping Chen

**Affiliations:** ^1^ School of Chinese Medicine LKS Faculty of Medicine The University of Hong Kong Hong Kong China; ^2^ Shanghai Mental Health Center Shanghai Jiao Tong University School of Medicine Shanghai China; ^3^ The Second Affiliated Hospital of Guangxi University of Chinese Medicine Nanning Guangxi China; ^4^ Sichuan Neautus Traditional Chinese Medicine Co., Ltd. Chengdu China

**Keywords:** longgu, metal ions, multi‐target therapy, psychiatric disorders, synergistic network

## Abstract

Longgu (fossilized bone), a mineral medicine in traditional Chinese medicine, has been used for centuries to treat symptoms resembling modern anxiety and insomnia. This review evaluates the hypothesis that Longgu's therapeutic effects arise from a synergistic network of its constituent metal ions. We synthesized evidence from chemical analyses, pharmacological studies of Ca, Mg, Zn, Fe, Cu, and Mn, and clinical trials of Longgu‐containing formulations. The metal ions form an integrated network exhibiting three emergent properties. First, ionostatic balance is achieved through Ca/Mg‐mediated regulation of neuronal excitability. Second, cross‐pathway synergy occurs where zinc concurrently modulates neurotransmission and promotes neuroplasticity via BDNF. Third, systemic integration involves zinc and manganese co‐regulating the gut–brain axis. Clinical studies demonstrate that Longgu‐containing formulations improve outcomes in depression, anxiety, and insomnia, particularly for comorbid conditions, and show synergistic effects when combined with conventional antidepressants. This network pharmacology framework repositions Longgu as a model for developing multi‐target therapeutics, offering a strategy to overcome limitations of single‐target psychiatric drugs.

## Graphical abstract

Longgu (fossilized bone) delivers a natural synergistic network of metal ions, Ca^2^^+^, Mg^2^^+^, Zn^2^^+^, Fe, Cu, and Mn^2^^+^ that collectively targets the interconnected pathologies of anxiety, depression, and insomnia. This multi‐ion system operates through coordinated mechanisms including ionostatic balance, cross‐pathway synergy, and systemic integration, offering a blueprint for multi‐target therapy in psychiatric disorders.

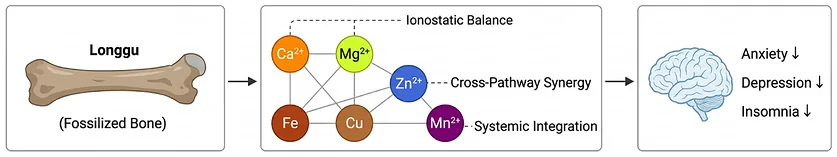

## 1 Introduction

Psychiatric disorders constitute a major global health challenge. The 2019 Global Burden of Disease Study reported that approximately 970 million people worldwide experience mental disorders. This figure has increased due to recent socioeconomic pressures [1]. Recent trend analyses have further documented rising anxiety disorders among adolescents [2] and highlighted social determinants such as education and income as critical factors influencing mental health outcomes [3]. Beyond individual suffering, these conditions impose substantial economic costs, with projected productivity losses of US $16.3 trillion between 2011 and 2030 [4]. These statistics underscore the urgent need for more effective and accessible treatments. However, current psychiatric drug development, largely based on the single‐target paradigm established in the mid‐20th century, has not adequately met this need.

Selective serotonin reuptake inhibitors (SSRIs) remain first‐line treatments for depression and anxiety disorders. Despite their clinical utility, their limitations are increasingly recognized. Clinical studies indicate that only 30%–40% of patients with major depressive disorder achieve remission with initial SSRI treatment, leaving many with residual or treatment‐resistant symptoms [5, 6]. Additionally, approximately 50% of patients experience adverse effects including nausea, sexual dysfunction, weight gain, and insomnia, which substantially affect treatment adherence [7]. Similar patterns occur with other psychiatric medications; atypical antipsychotics, for example, frequently cause metabolic disturbances and sedation [8]. Psychotherapy, while effective for many patients, faces implementation barriers including limited access to trained providers and variable patient engagement [9, 10].

The limited success of single‐target approaches reflects a fundamental disconnect with the complex biology of psychiatric disorders. Rather than resulting from simple “chemical imbalances,” conditions such as depression, anxiety, and schizophrenia involve dysregulation across multiple interconnected systems. Many patients present with comorbid conditions, such as depression with anxiety or insomnia with cognitive impairment, that single‐target drugs cannot adequately address [11]. The high prevalence of psychiatric comorbidities is well‐documented across diverse clinical populations, including individuals with epilepsy [12] and those with physical injuries [13].

Current pathophysiological understanding recognizes several contributing factors. These include imbalances in multiple neurotransmitter systems (serotonin, dopamine, glutamate, and GABA). Chronic neuroinflammation is marked by elevated cytokines including interleukin‐6 and tumor necrosis factor‐α [14]; Impaired neuroplasticity is evidenced by reduced brain‐derived neurotrophic factor (BDNF) levels and decreased dendritic spine density [15, 16]. Additionally, gut–brain axis dysfunction occurs where altered microbiota and increased intestinal permeability promote systemic inflammation [17, 18]. This complex network of pathological processes renders conventional single‐target interventions inadequate.

Traditional Chinese Medicine (TCM) offers an alternative conceptual framework based on multi‐component, multi‐target therapeutic strategies. TCM formulations typically combine multiple herbs and minerals to achieve systemic regulation rather than single‐pathway inhibition. Longgu (fossilized bone) occupies a distinctive position within this tradition.

First documented in the Shennong Bencao Jing as a “superior‐grade” medicine for “calming the spirit and pacifying the liver,” it has been used for symptoms now recognized as anxiety, insomnia, and palpitations [19]. Its continued use in classical formulas such as Chaihu Jia Longgu Muli Tang and Guizhi Gancao Longgu Muli Tang attests to its perceived therapeutic value [20, 21].

Modern analytical methods have characterized Longgu's material composition. It consists primarily of hydroxyapatite [Ca_10_(PO_4_)_6_(OH)_2_], which serves as a natural reservoir for multiple metal ions [22, 23]. The composition includes calcium (30%–40%) and phosphorus (15%–20%), along with neuroactive trace elements including magnesium (0.2%–0.5%), zinc, iron (0.01%–0.4%), copper, and manganese (0.001%–0.04%) [23, 24, 25]. Previous research has largely focused on individual ions: calcium triggers neurotransmitter release, and synaptic plasticity [26, 27]; magnesium blocks NMDA receptors to prevent excitotoxicity [28, 29]; zinc modulates GABA and glutamate transmission and regulates BDNF [30, 31]; iron and copper serve as cofactors for monoamine synthesis [32, 33]. However, this reductionist approach overlooks potential interactions among these elements.

In this review, we propose and evaluate the hypothesis that Longgu's therapeutic effects emerge from synergistic interactions among its constituent metal ions, forming what we term a “natural synergistic network.” We suggest these ions function collectively as an integrated system with three core properties: ionostatic balance through calcium–magnesium coordination; cross‐pathway synergy exemplified by zinc's dual effects on neurotransmission and neuroplasticity; and systemic integration through zinc–manganese modulation of the gut–brain axis. This framework repositions Longgu as a potential model for multi‐target therapeutic development. We synthesize evidence from chemical analysis, molecular pharmacology, animal studies, and clinical trials to evaluate this hypothesis and outline a research roadmap for translating these insights into modern therapeutic strategies.

## 2 Deconstructing the Blueprint: The Composition and Innate Synergies of Longgu's Ion Network

To comprehend the therapeutic sophistication of Longgu, one must first move beyond viewing it as a mere mineral and instead appreciate it as a complex, naturally engineered delivery system. Its efficacy is not a product of a single active compound but emerges from the intricate matrix that hosts a specific profile of bioavailable elements. This chapter deconstructs this blueprint, detailing the material foundation of Longgu and the intrinsic, pre‐programmed synergies between its core metal ions that form the basis of its network pharmacology.

### 2.1 A Complex Mineral Matrix: More Than a Simple Source

The primary architectural foundation of Longgu is hydroxyapatite [Ca_10_(PO_4_)_6_(OH)_2_], the principal mineral component of vertebrate skeletons [22, 23]. However, characterizing Longgu as merely fossilized bone overlooks its unique geochemical evolution. The hydroxyapatite structure undergoes dynamic isomorphic substitution, an active process whereby calcium ions in the crystal lattice are replaced by cations of similar ionic radius. Examples include strontium (Sr), sodium, or rare Earth elements (REEs). Concurrently, phosphate groups can be substituted by vanadium or silicon, and hydroxyl groups by fluorine [34, 35, 36, 37]. This process yields a complex, doped mineral system whose final elemental composition reflects its depositional environment [37]. This inherent complexity provides the first indication that Longgu's bioactivity is fundamentally multi‐elemental.

Elemental analyses via techniques such as inductively coupled plasma mass spectrometry (ICP‐MS) have quantified this diversity, revealing a composition more complex than that of modern bone. While calcium (30%–40%) and phosphorus (15%–20%) constitute the bulk material, the trace element profile defines Longgu's distinctive character [23]. This profile includes essential neuroactive metals: iron (Fe, 0.01%–0.4%), magnesium (Mg, 0.2%–0.5%), zinc (Zn), copper (Cu), and manganese (Mn, 0.001%–0.04%) [23, 24]. Specific elements serve as markers of authenticity and bioactivity; for instance, strontium (Sr > 1200 ppm) and fluorine (F > 700 ppm) concentrations in genuine Longgu significantly exceed those in modern bones (typically 300–800 ppm), providing a chemical fingerprint for quality control [37]. Furthermore, the total REE content correlates with geological age. Samples dated to 43 500 years exhibit higher REE levels than younger counterparts. This suggests that the duration of fossilization critically influences the mineral's bioactivity and ion reservoir stability [23].

This composition is not static but is profoundly shaped by two key factors: geographical origin and traditional processing. The concept of *Dao Di* in TCM, which emphasizes the superior quality of herbs from specific regions, is chemically validated in Longgu. Pronounced geographical disparities in its elemental profile have been documented. For instance, Longgu from Fugu, Shaanxi, contains the highest levels of Mn. These measure 1.8 to 129 times higher than samples from other regions. Samples from Fengrun and Nihewan in Hebei province are notably rich in Zn, with concentrations reaching 2040 ppm. This is 32 times that of Longgu from Chifeng, Inner Mongolia, and 107 times that from Xia County, Shanxi province [25]. The *Wuhua Longgu* from Yushe, Shanxi, is characterized by exceptionally high aluminum (Al, 11 700 ppm) and Mn content [25]. These geographical signatures are not merely academic; they likely underpin the observed variations in the sedative and anxiolytic efficacy of Longgu from different sources, implying that the specific “flavor” of the synergistic network can vary.

Recent geochemical analyses have further elucidated the relationship between fossilization duration and elemental enrichment. Studies employing x‐ray fluorescence and molecular structure analysis demonstrate that strontium incorporation into the hydroxyapatite lattice follows predictable patterns based on burial conditions and geological age [38, 39, 40]. These findings provide a scientific basis for understanding why Longgu from different deposits may exhibit varying therapeutic properties. Structural analysis of Longgu samples from different geographical origins has revealed distinct crystallographic features corresponding to their elemental profiles. X‐ray diffraction studies show that variations in trace element content, particularly strontium and manganese, are reflected in the lattice parameters of the hydroxyapatite structure [40]. These structural differences may influence the release kinetics of ions during decoction preparation.

Furthermore, processing (*Paozhi*) is a deliberate, traditional intervention designed to optimize the network's activity. The primary method, calcination (*Duanzhi*), is not a simple sterilization step but a transformative process that alters the mineral composition and enhances ion bioavailability. Calcination drives off volatile organic compounds and converts carbonates. This leads to a material with a higher total Ca content (95.8%–101.1% in calcined vs. 89.1%–95.7% in raw Longgu) and a more porous, reactive structure [41, 42]. Research indicates that the optimal calcination temperature lies between 750°C and 800°C, a range that maximizes this transformation while preserving the structural integrity of the ion‐hosting matrix [42, 43]. This process increases the extraction efficiency of components in decoctions, thereby enhancing the concentration of bio‐accessible ions like Ca^2^^+^ that are ready for physiological interaction [44, 45]. In essence, traditional processing can be viewed as a form of ancient pharmaceutical engineering that fine‐tunes the release kinetics and bioavailability of the active ion network.

The structural changes occurring during Longgu calcination have been characterized using molecular spectroscopic techniques. Research shows that heating induces progressive decomposition of organic residues and recrystallization of the mineral matrix, with optimal structural reorganization occurring between 750°C and 800°C [39]. These temperature‐dependent changes directly influence the bioavailability of calcium and other ions in aqueous decoctions. The elemental composition of Longgu and the factors influencing it, such as geographical origin and processing, are summarized in Table 1.

**TABLE 1:** Metal element composition of Longgu and influencing factors [23].

Category	Origin/processing method	Key elements & changes	Comparison/data/implications
Elemental composition (Major)	—	Ca, P	‐ Core Elements: Ca (30%–40%), P (15%–20%). High Ca/P ratio (>2) due to fossilization. ‐ Association: High Fe indicates higher petrifaction; high Mg may relate to burial environment.
Elemental composition (Trace & Characteristic)	—	Sr, F, Zn, Al, and Mn	‐ Authentication: Sr >1200 ppm, F >700 ppm (significantly higher than modern bones). ‐ Characteristic: *Wuhua Longgu* has high Mn & Al. ‐ Enrichment/Depletion: Sr, Ba, U are enriched; Sc, V, Cr, Co, Ni, and Cu are generally depleted.
Geographical influence	Fugu, Shaanxi	Highest Mn content	1.8–129 times higher than other origins.
	Fengrun / Nihewan, Hebei	Significantly high Zn content	2040 ppm; 32x Chifeng, 107x Xia County.
	*Wuhua Longgu*, Yushe, Shanxi	High Al and Mn content	Al: 11 700 ppm (1.3x Baode, 37x Shenmu).
	Nihewan	Relatively high Se content	Most samples only 1/10 of Nihewan's content.
Processing effects	Calcination (*Duanzhi*)	Increases Ca content, alters mineral composition	Calcined: Ca 95.8%–101.1%; Raw: 89.1%–95.7%.
	Calcination Temperature	Decomposition of volatile substances	Optimal range: 750°C–800°C to preserve properties.

### 2.2 The Core Components of the Network and Their Primary Functions

Within this optimized mineral matrix resides the functional unit of Longgu's action: a consortium of metal ions, each playing a distinct yet deeply interconnected role in neuronal regulation. Understanding their individual part is a prerequisite for appreciating the symphony of their interactions.

#### 2.2.1 Ca and Mg: The “Ionostatic Duo” Regulating Neuronal Excitability

Calcium ions (Ca^2^^+^) are fundamental to neuronal signaling. Their influx through presynaptic voltage‐gated calcium channels (VGCCs) is the essential trigger for synaptic vesicle fusion and the release of neurotransmitters such as glutamate and dopamine [26, 27]. Post‐synaptically, Ca^2^^+^ functions as a critical second messenger. It binds to calmodulin (CaM) to activate enzymes including Ca^2^^+^/calmodulin‐dependent protein kinase II (CaMKII) and protein kinase C (PKC). These enzymes regulate gene expression underlying synaptic plasticity and memory formation [46, 47]. The genetic association between VGCC dysfunction, particularly variants in the *CACNA1C* gene, and disorders like schizophrenia and bipolar disorder, underscores the central pathophysiological role of Ca^2^^+^ [48, 49]. In contrast, magnesium (Mg^2^^+^) serves as an endogenous brake on excitation. It acts as a voltage‐dependent blocker of NMDA receptors and certain VGCCs, occupying the channel pore at resting potentials to prevent pathological Ca^2^^+^ influx and excitotoxicity [28, 29]. This inherent functional antagonism, Ca^2^^+^ as the accelerator and Mg^2^^+^ as the brake, constitutes the network's primary mechanism for maintaining neuronal excitability within a homeostatic range. The clinical relevance of this balance is underscored by Mg deficiency inducing anxiety‐like behaviors and HPA axis dysregulation in animals. Additionally, an inverse correlation exists between serum Mg levels and depression severity in humans [50, 51, 52].

**FIGURE 1:**
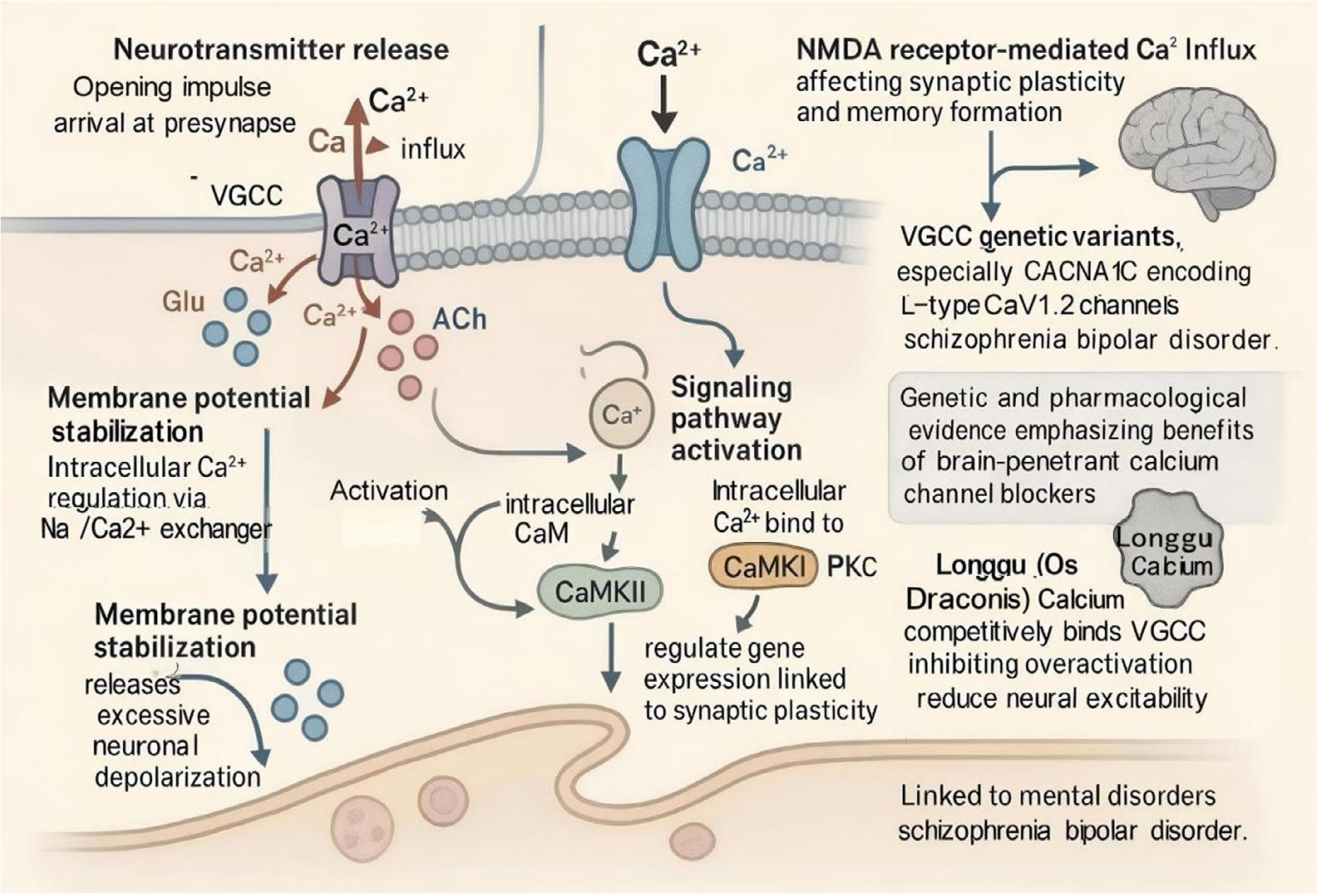
Calcium signaling in neurons and potential modulation by Longgu. Calcium influx through VGCCs triggers neurotransmitter release and activates CaM/CaMKII/CREB pathways regulating synaptic plasticity. Genetic variants in VGCC subunits (e.g., CACNA1C) are associated with psychiatric disorders. Longgu‐derived calcium may help stabilize aberrant neuronal excitability. Abbreviations: CaM, calmodulin; CaMKII, Ca^2^^+^/calmodulin‐dependent protein kinase II; CREB, cAMP response element‐binding protein; VGCC, voltage‐gated calcium channel.

**FIGURE 2:**
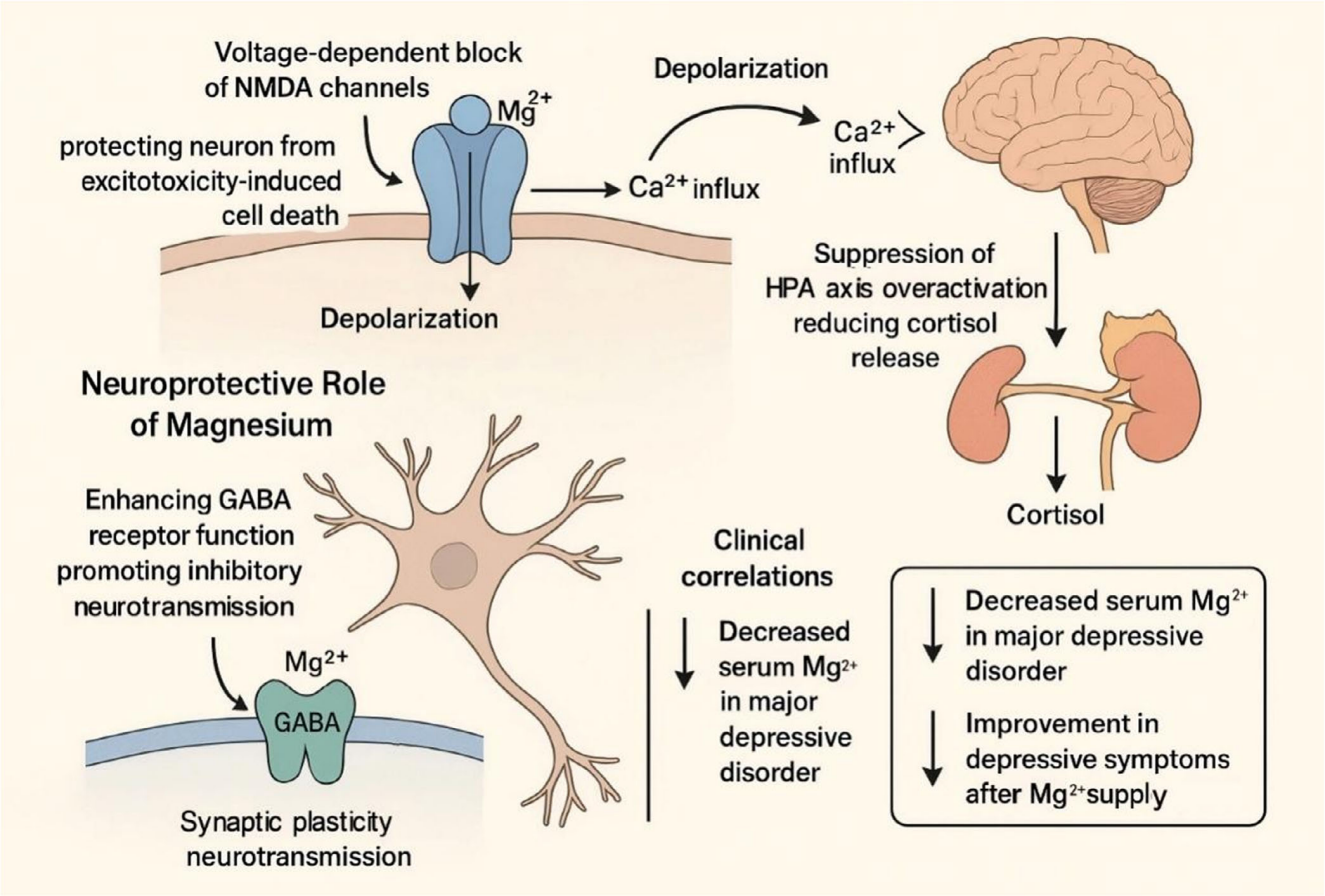
Neuroprotective mechanisms of magnesium and clinical relevance to depression. Magnesium blocks NMDA receptors in a voltage‐dependent manner, enhances GABAergic inhibition, and regulates HPA axis activity. Clinical studies show reduced serum magnesium in depressed patients, with severity correlating inversely with magnesium levels. Abbreviations: GABA, gamma‐aminobutyric acid; HPA, hypothalamic–pituitary–adrenal; MDD, major depressive disorder; NMDA, N‐methyl‐D‐aspartate; PHQ‐9, Patient Health Questionnaire‐9.

The coordination chemistry of calcium with biologically active ligands provides insights into how calcium ions might interact with endogenous molecules to produce therapeutic effects. Studies of calcium complexes with anti‐inflammatory agents demonstrate that Ca(II) coordination can modify the pharmacological properties of organic compounds, potentially enhancing their bioavailability or target specificity [53]. This raises the possibility that calcium in Longgu may similarly interact with organic components in TCM formulations to modulate its overall therapeutic profile. A schematic summarizing the central role of calcium ions in neuronal signaling and the proposed mechanism of Longgu is provided in Figure 1. Also, the multifaceted neuroprotective roles of magnesium and their clinical correlation with depression are illustrated in Figure 2.

#### 2.2.2 Zn: The “Central Integrator” Linking Synaptic Function to Plasticity

**FIGURE 3:**
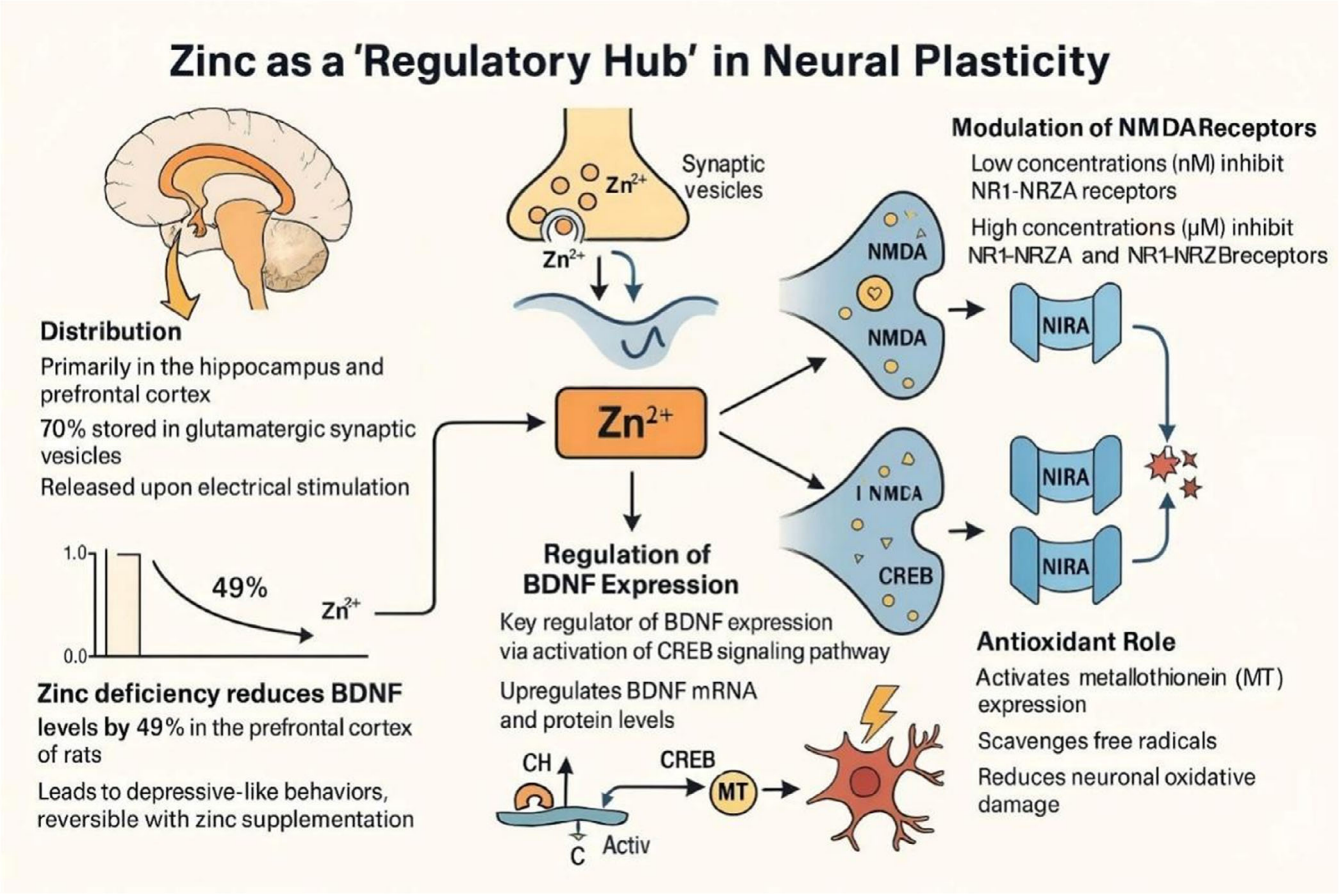
Zinc as a central integrator of neurotransmission and neuroplasticity. Synaptically released zinc modulates NMDA and GABA receptors to regulate excitatory–inhibitory balance. Intracellular zinc activates CREB signaling, increasing BDNF expression and promoting synaptic plasticity via TrkB/PI3K/Akt and MAPK pathways. Zinc also induces metallothionein for antioxidant defense. Zinc deficiency reduces BDNF and produces depression‐like behaviors. Abbreviations: BDNF, brain‐derived neurotrophic factor; CREB, cAMP response element‐binding protein; MAPK, mitogen‐activated protein kinase; MT, metallothionein; PI3K/Akt, phosphoinositide 3‐kinase/protein kinase B; TrkB, tropomyosin receptor kinase B.

Zinc exemplifies multi‐target regulation within the network. It is highly concentrated in the synaptic vesicles of hippocampal and prefrontal glutamatergic neurons and is co‐released with glutamate upon activation [30]. Synaptic Zn^2^^+^ acts as a potent, concentration‐dependent modulator. It inhibits NMDA receptors via high‐affinity binding to the GluN2A subunit, reducing channel open probability and protecting against excitotoxicity [54, 55]. Concurrently enhancing GABAergic inhibition by increasing interneuron excitability and GABA release [56, 57]. This bidirectional fine‐tuning of excitatory‐inhibitory balance is complemented by Zn's role as a master regulator of neuroplasticity. Zinc upregulates BDNF expression by activating the CREB signaling pathway [31]. The subsequent activation of the BDNF/TrkB axis promotes dendritic spine formation, synaptogenesis, and neuronal survival via downstream PI3K/Akt and MAPK signaling cascades [58, 59]. The critical importance of this role is demonstrated in animal models, where Zn deficiency induces a 49% reduction in frontal cortex BDNF levels and depression‐like behaviors, both reversible upon Zn supplementation [60]. Recent advances in understanding metal‐based neuroprotection have revealed that zinc operates through multiple coordinated pathways. A systematic review of metal‐containing compounds in traditional medicine demonstrates that zinc complexes exhibit neuroprotective effects through mechanisms including oxidative stress reduction, mitochondrial stabilization, and modulation of apoptotic signaling [61]. These findings support the conceptualization of zinc as part of an integrated metal network rather than an isolated therapeutic agent. Thus, Zn serves as a central hub, integrating the fast, ion‐mediated regulation of synaptic transmission with the slower, trophic processes of structural remodeling. The role of zinc as a central regulatory hub coordinating neurotransmission and neuroplasticity is summarized in Figure 3.

#### 2.2.3 Fe and Cu: The “Metabolic Engines” of Monoamine Neurotransmission

Iron and copper are indispensable for the synthesis and metabolism of monoamine neurotransmitters central to mood, motivation, and attention. Iron serves as a critical cofactor for the rate‐limiting enzymes tyrosine hydroxylase and tryptophan hydroxylase. These enzymes are essential for dopamine and serotonin production, respectively [32, 62]. Consequently, iron deficiency directly impairs the synthesis of these neurotransmitters, manifesting as low mood, fatigue, and poor concentration. This is clinically relevant; children with attention‐deficit/hyperactivity disorder exhibit significantly lower serum ferritin, and iron supplementation can achieve a 62.3% rate of symptom improvement [63, 64].

Copper plays an equally pivotal role in catecholamine conversion and catabolism. It is an essential component of dopamine β‐hydroxylase, which catalyzes the conversion of dopamine to norepinephrine. Thus, copper shapes the catecholamine balance [33, 65]. Copper also influences monoamine oxidase activity, the primary enzyme responsible for monoamine degradation [65]. This dual function positions copper as a key regulator of neurotransmitter turnover. The coordination chemistry of copper in the central nervous system has been extensively characterized. Recent studies demonstrate that copper ions bind to specific protein sites involved in neurotransmitter synthesis and degradation, with the redox activity of copper playing a critical role in maintaining proper catecholamine balance [66]. This precise coordination chemistry explains why both copper deficiency and excess can disrupt monoaminergic neurotransmission. However, both ions exemplify the network's requirement for precise homeostasis, as dysregulation is detrimental. Iron overload catalyzes destructive reactive oxygen species (ROS) via the Fenton reaction, promoting oxidative stress and neuroinflammation [67, 68], while both deficiency and excess of copper are implicated in schizophrenia, bipolar disorder, and depression [69].

#### 2.2.4 Mn: The “Mitochondrial and Microbial Modulator”

Manganese functions at the nexus of cellular energy metabolism and gut–brain communication. As a critical component of the mitochondrial antioxidant enzyme Mn‐superoxide dismutase (Mn‐SOD), it protects neural cells from oxidative damage [70]. Manganese also supports mitochondrial respiratory chain function, thereby contributing to efficient neuronal energy production. Its therapeutic window is narrow; however, as excessive accumulation is neurotoxic [71]. The moderate Mn content in Longgu (0.003%–0.008%) is therefore likely essential for conferring benefit without risk [23]. A novel and significant role for manganese involves its influence on the gut microbiota. Manganese ions are essential for commensal bacteria, such as *Lactobacillus*, to defend against oxygen toxicity. Adequate Mn levels promote their proliferation [72]. Given that specific intestinal *Lactobacillus* strains stimulate GABA production and the gut is a major source of peripheral GABA, Mn indirectly supports a peripheral reservoir of this inhibitory neurotransmitter. Gut‐derived GABA is postulated to relay calming signals to the brain via the vagus nerve [73]. This positions Mn as a unique contributor to the network's systemic reach.

The therapeutic innovation of Longgu resides not in the mere presence of its individual ions, but in their complex web of interactions. The network's efficacy is embedded in documented synergistic and antagonistic relationships, detailed in Table 2. For instance, Zn and Mg act synergistically to enhance neurite outgrowth and the formation of complex neuronal networks, suggesting a cooperative role in promoting neuroplasticity [45]. In contrast, the antagonistic relationship between Ca and Mg, where neurotransmitter release is directly proportional to extracellular Ca^2^^+^ and inversely proportional to Mg^2^^+^, creates a built‐in, self‐limiting system for fine‐tuning synaptic strength [74]. Furthermore, Zn and Ca exhibit both competitive and synergistic behaviors, vying for cellular transporters while also co‐regulating synaptic plasticity, indicating a complex homeostatic relationship essential for optimal neuronal function [75]. This intricate web of interactions, the natural synergistic network, ensures a coordinated, multi‐front intervention beyond the capability of any single ion or single‐target drug. The role of zinc as a central hub is summarized in Figure 3. It is this emergent system, deconstructed here in its composition and innate synergies, whose therapeutic actions across psychiatric disease pathologies, we will elucidate in the following chapter.

**TABLE 2:** Documented synergistic and antagonistic interactions between key metal ions in Longgu.

Ion pair	Type of interaction	Biological context & effect	Implication for Longgu's action	References
**Zn & Mg**	**Synergistic**	Co‐enhance neurite outgrowth and formation of advanced neuronal networks.	May underpin Longgu's role in promoting neuroplasticity and cognitive improvement.	[45]
**Ca & Mg**	**Antagonistic**	Mg^2^^+^ inhibits VGCCs, limiting Ca^2^^+^ influx and preventing excessive neurotransmitter release.	Creates a built‐in regulatory mechanism for fine‐tuning neuronal excitability, preventing overstimulation.	[76, 115]
**Zn & Ca**	**Competitive/Synergistic**	Compete for transporters; yet synergistically regulate synaptic plasticity.	Suggests a complex, homeostatic relationship crucial for maintaining synaptic balance.	[75]
**Fe & Cu**	**Metabolic Interplay**	Both are essential for oxidative metabolism; imbalance in one can affect the other (e.g., in ceruloplasmin function).	The moderate levels of both in Longgu may help maintain metabolic balance in neural cells.	[116]

## 3 The Emergent Properties of the Synergistic Network in Action

**FIGURE 4:**
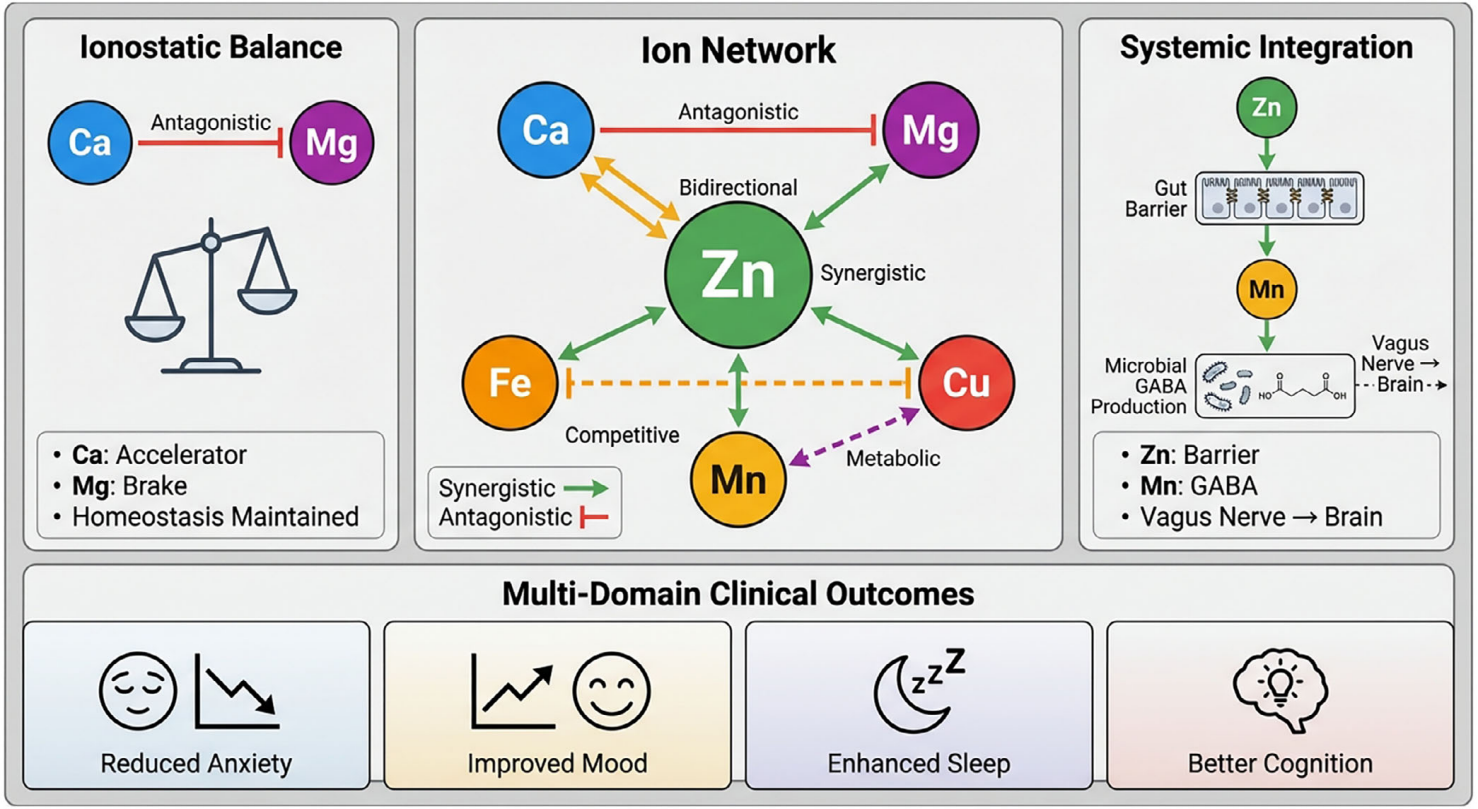
The natural synergistic network of metal ions in Longgu. The central panel shows the six core ions and their documented interactions. Surrounding panels illustrate the three emergent properties: Ionostatic Balance through Ca/Mg coordination of neuronal excitability; Cross‐Pathway Synergy where zinc modulates both neurotransmission and BDNF‐mediated plasticity; and Systemic Integration through Zn/Mn regulation of gut barrier function and microbial GABA production. Bottom panel indicates the multi‐domain clinical outcomes resulting from network activity.

The therapeutic sophistication of Longgu is demonstrated not by cataloging its individual components, but by the system‐level behaviors emerging from their interactions. Emergent properties, capabilities arising from interactions that individual parts lack, define a network's function. When Longgu's metal ions engage the complex pathophysiology of psychiatric disorders, they function not as discrete agents but as a coordinated unit, exhibiting three core emergent properties: ionostatic balance, cross‐pathway synergy, and systemic integration. This section elucidates the collective action of this natural network, demonstrating its concurrent stabilization, repair, and rebalancing of the dysregulated systems underlying mental illness. The proposed natural synergistic network and its three core emergent properties are illustrated schematically in Figure 4, which provides a visual framework for understanding how the six metal ions interact to produce multi‐domain therapeutic effects.

### 3.1 Emergent Property 1: Ionostatic Balance—The Network's Built‐In Stabilizer

A fundamental requirement of nervous system health is the maintenance of neuronal excitability within a stable, homeostatic range. Pathological states such as anxiety and mania are frequently characterized by regulatory failure, leading to neuronal hyperexcitability. The Longgu network addresses this dysregulation not via blanket suppression, but through a dynamic, self‐adjusting mechanism termed ionostatic balance. This mechanism is primarily orchestrated by the Ca/Mg duo.

This balance operates on a principle of inherent functional antagonism. Calcium serves as the primary driver of neuronal excitation. A nerve impulse arriving at the presynaptic terminal triggers VGCC opening, permitting Ca^2^^+^ influx that is essential for synaptic vesicle fusion and the release of neurotransmitters such as glutamate, thereby propagating the signal [26, 27, 76]. Post‐synaptically, Ca^2^^+^ influx, particularly through NMDA receptors, initiates signaling cascades critical for synaptic plasticity. However, excessive excitatory drive can lead to excitotoxicity, a process implicated in neuronal damage observed in severe stress, anxiety, and neurodegenerative conditions [77, 78].

Magnesium serves as the network's endogenous, voltage‐dependent brake. At resting membrane potentials, Mg^2^^+^ ions physically occupy the pores of NMDA receptors and certain VGCCs, establishing a tonic blockade that prevents aberrant Ca^2^^+^ entry [28, 29, 76]. Only upon sufficient neuronal depolarization is the Mg^2^^+^ block expelled, permitting controlled Ca^2^^+^ influx required for physiological processes like long‐term potentiation (LTP) [29]. This constitutes a graded, dynamic damping system rather than a simple binary switch. The concurrent presence of both ions establishes a stable system resistant to runaway excitation.

The pathological relevance of Ca/Mg balance is demonstrated in experimental anxiety models. Artificially induced Mg deficiency in mice produces robust anxiety‐like behaviors, HPA axis dysregulation, and neuronal hyperexcitability in the hypothalamic paraventricular nucleus, a key stress center [50, 51]. This Mg‐deficient state effectively disables the network's brake, permitting unchecked excitatory activity. Clinically, this is mirrored by significantly lower serum Mg levels in patients with major depressive disorder, showing a negative correlation with depression severity [52].

The concurrent presence of bioavailable Ca and Mg in Longgu is thus pharmacologically profound, providing a natural reset mechanism. Instead of non‐specifically suppressing or stimulating neuronal activity, the network supplies the ions required to restore critical equilibrium. Bioavailable Mg reinstates the voltage‐dependent block on overactive NMDA receptors and VGCCs, while Ca ensures fundamental signaling and plasticity processes remain supported. Ionostatic balance is therefore the network's primary emergent property: a self‐regulating system that stabilizes neuronal firing and prevents the pathological excitability swings underlying numerous psychiatric symptoms. This ionostatic balance, mediated by the antagonistic relationship between calcium and magnesium, is represented in the left panel of Figure 4.

### 3.2 Emergent Property 2: Cross‐Pathway Synergy—The Network's Multi‐Target Engineer

A principal limitation of single‐target drugs is their failure to address interconnected pathological pathways. An agent that dampens neuronal hyperexcitation may not repair the resulting synaptic deficits. The Longgu network overcomes this via cross‐pathway synergy. This property is epitomized by Zn, which functions as a central integrator coordinating interventions across disparate pathological domains.

Zinc's role in fast synaptic transmission is well‐established. Co‐released with glutamate, it exerts concentration‐dependent, bidirectional regulation on NMDA receptors: at low nanomolar concentrations it selectively inhibits NR1–NR2A subtypes. At higher micromolar concentrations it broadly inhibits both NR1–NR2A and NR1–NR2B receptors, providing fine‐tuned control of excitatory currents and preventing excitotoxicity [54, 55]. Concurrently, in regions like the amygdala, Zn enhances GABAergic inhibition, promoting a calmer neuronal state [56, 57]. This immediate, ion‐mediated action directly counteracts dysfunctional neurotransmission.

The network's ingenuity, however, lies in the subsequent actions of the same Zn^2^^+^ ions. Beyond synaptic modulation, zinc acts as a potent upstream activator of the BDNF pathway. It upregulates BDNF expression by activating the CREB signaling pathway [50]. The resulting mature BDNF binds to its TrkB receptor, triggering downstream PI3K/Akt and MAPK cascades essential for dendritic spine formation, synaptogenesis, and neuronal survival [58, 59]. Thus, zinc addresses the critical domain of impaired neuroplasticity, a hallmark of depression characterized by neuronal atrophy and reduced spine density in the hippocampus and prefrontal cortex [15, 16].

The mechanistic link between zinc and BDNF has been further elucidated in recent pharmacological studies. Experimental evidence demonstrates that zinc‐induced BDNF expression involves activation of the TrkB receptor and subsequent phosphorylation of ERK1/2 and CREB, pathways that are essential for synaptic plasticity and neuronal survival [79]. This zinc‐BDNF axis represents a critical node through which the Longgu network couples immediate neurotransmitter modulation with longer‐term structural remodeling. Thus, a single network component, Zn, executes a coordinated, two‐pronged strategy: it first corrects functional signaling by modulating NMDA and GABA receptors to calm excessive firing, and then initiates structural repair by activating BDNF to rebuild synapses. This is cross‐pathway synergy in action. It ensures that immediate symptom relief is coupled with long‐term restoration, a capability absent in drugs targeting a single transporter or receptor. This synergy extends beyond Zn; Ca^2^^+^, while driving excitation, also activates CaMKII and CREB pathways to regulate gene expression required for synaptic remodeling [46, 47, 80], demonstrating the network's design principle of multi‐functional components whose actions concurrently influence multiple pathological domains. A comparative analysis of conventional single‐target drugs and Longgu's multi‐target approach, detailing the fundamental roles of its core ions, is presented in Table 3. Zinc's function as a central integrator, coordinating both fast synaptic transmission and longer‐term neuroplasticity, is illustrated in the central hub position of Figure 4, with connections radiating to all other ions.

**TABLE 3:** Comparative analysis of therapeutic approaches and fundamental properties of Longgu's core metal ions.

Feature	Conventional single‐target drugs (e.g., SSRIs)	Longgu's multi‐ion, multi‐target approach (Proposed)	Core metal ions & their fundamental roles
Primary mechanism	Single target (e.g., Serotonin Transporter).	Multiple targets simultaneously (VGCCs, NMDARs, GABAAR, Nrf2, BDNF, etc.).	Ca: “Switch Molecule” for neuronal signaling; triggers release, stabilizes potential, activates plasticity genes. Mg: “Regulatory Factor” in neuronal inhibition; blocks NMDA, enhances GABA, inhibits HPA axis. Zn: “Regulatory Hub” of neuroplasticity; modulates GABA/NMDA, upregulates BDNF, antioxidant. Fe: “Essential Cofactor” for monoamine synthesis (DA, 5‐HT); imbalance (deficiency/overload) affects mood & cognition. Cu & Mn: “Balancing Factors” in neural metabolism; Cu for NE synthesis/redox balance, Mn for antioxidant defense/energy.
Efficacy in comorbidity	Limited; often requires polypharmacy.	High potential; inherently designed to address co‐occurring symptoms.	
Common side effects	Nausea, sexual dysfunction, weight gain, insomnia.	Expected to be lower due to physiological ion levels and homeostatic antagonism (e.g., Ca^2^^+^/Mg^2^^+^ balance).	
Therapeutic onset	4–6 weeks for full effect.	Potentially faster for some symptoms (e.g., acute calming), but requires study.	
Scientific basis	Reductionist, target‐driven.	Holistic, systems biology‐driven, based on natural ion synergy.	

Abbreviations: 5‐HT, serotonin; BDNF, brain‐derived neurotrophic factor; Ca, calcium; Cu, copper; DA, dopamine; Fe, iron; GABA, gamma‐aminobutyric acid; GABAAR, GABA‐A receptor; HPA, hypothalamic‐pituitary‐adrenal; Mg, magnesium; Mn, manganese; NE, norepinephrine; NMDAR, N‐methyl‐D‐aspartate receptor; Nrf2, nuclear factor erythroid 2‐related factor 2; SSRI, selective serotonin reuptake inhibitor; VGCC, voltage‐gated calcium channel; Zn, zinc.

### 3.3 Emergent Property 3: Systemic Integration—The Network's Holistic Regulator

Perhaps the most forward‐thinking emergent property is systemic integration. The brain does not operate in isolation. The network extends its regulatory influence to the gut–brain axis, a major pathway in psychiatric pathophysiology. This property is driven by the complementary actions of Zn and Mn, which together orchestrate a holistic intervention from the intestine to the central nervous system.

The gut–brain axis is a bidirectional communication network where the intestinal environment profoundly influences brain function. Psychiatric disorders are frequently associated with increased intestinal permeability (“leaky gut”) and dysbiosis, which can drive systemic inflammation that exacerbates neuroinflammation [17, 18]. Zinc serves as a key guardian of intestinal integrity by promoting the expression of tight junction proteins, including occludin, zonula occludens‐1 (ZO‐1), and claudin‐3. This reinforces the gut barrier and reduces translocation of pro‐inflammatory molecules such as lipopolysaccharide (LPS) into circulation [81]. Furthermore, Zn supplementation corrects dysbiosis, increasing the abundance of beneficial bacteria (e.g., *Bifidobacterium*, *Lactobacillus*) while inhibiting pathogens such as *Escherichia coli* [82]. This is clinically relevant, as Zn deficiency alters the pediatric gut microbiota, an effect reversible by Longgu preparations and correlated with reduced anxiety‐like behaviors [83, 84, 85]. Recent mechanistic studies have further elucidated how gut microbiota‐derived metabolites, particularly short‐chain fatty acids, influence depressive symptoms through immune and neuroendocrine pathways [86]. Mendelian randomization analyses have also revealed causal associations between gut microbiota composition and specific depressive symptom profiles [87]. The gut–brain axis represents a particularly promising target for metal‐based therapeutics. Emerging evidence indicates that the gut microbiota responds to dietary metal ions in ways that influence central nervous system function through vagal and humoral pathways [58]. This research highlights how mineral composition can modulate the bidirectional communication between the intestinal environment and brain function, providing a mechanistic basis for the systemic effects observed with Longgu administration. Zinc and manganese show complementary effects on gut barrier function and microbial GABA production, respectively [88].

Manganese plays a distinct but equally vital role in this gut‐based strategy. If Zn fortifies the intestinal barrier, Mn arms the commensal allies. Manganese ions are essential for bacteria like *Lactobacillus* to defend against oxygen toxicity, and adequate Mn levels promote their proliferation [72]. This is critical because specific *Lactobacillus* strains potently stimulate GABA synthesis by intestinal cells [73]. As the gut is a major source of peripheral GABA, Mn‐supported proliferation of these bacteria can elevate intestinal GABA levels. This peripherally produced GABA is postulated to relay calming signals to the brain via the vagus nerve, exerting a systemic anxiolytic effect.

Thus, through systemic integration, the Longgu network executes a comprehensive gut‐brain strategy: it simultaneously fortifies the intestinal barrier (via Zn) to prevent a key inflammatory source, and enlists the microbiota (via Mn) to generate calming signals. This dual action concurrently reduces a pathological driver (LPS‐triggered neuroinflammation) and enhances a physiological regulator (vagal GABAergic tone). This emergent property demonstrates the network's reach beyond the synapse to engage the body's wider physiology, offering a holistic mechanism perfectly aligned with the multi‐system nature of psychiatric disorders. The core mechanisms by which Longgu's metal ions regulate key pathological domains in psychiatric disorders are synthesized in Table 4.

**TABLE 4:** Core mechanisms of metal ion regulation in psychiatric disorders.

Regulatory dimension	Involved metal ions	Core mechanisms	Associated psychiatric disorders/pathological states
**Neurotransmitter system regulation**	Ca, Mg, Zn, Fe, Cu	1. Ca–Mg Synergy: Ca triggers release, Mg inhibits Ca channels to limit excess release, maintaining balance. 2. Zn Bidirectional Regulation: Concentration‐dependent NMDA receptor inhibition; enhanced GABAergic transmission; balances excitation/inhibition. 3. Fe/Cu Metabolic Regulation: Fe promotes monoamine synthesis; Cu involved in DA‐to‐NE conversion and degradation.	Anxiety (Ca/Mg imbalance), Depression (Fe/Zn deficiency), Schizophrenia (Cu dysregulation → excess NE), Cognitive Impairment (Zn/NMDA system abnormality)
**Neuroinflammation intervention**	Zn, Fe, Mg, Cu	1. Imbalance Triggers: Fe overload → ROS → oxidative stress; Zn deficiency → NF‐κB activation → inflammation. 2. Anti‐inflammatory Pathway: Zn activates Nrf2/HO‐1 pathway, downregulating inflammatory proteins. 3. Bidirectional Regulation: Inflammatory cytokines affect metal metabolism (e.g., IL‐6 → Fe accumulation); metal ions modulate cytokine secretion.	Neurodegeneration (Fe overload), Depression (elevated cytokines), Epilepsy (NF‐κB mediated inflammation), Neural Injury (oxidative stress‐related)
**Neuroplasticity Regulation**	Zn, Ca, Cu	1. Structural Regulation: Zn promotes dendritic spine formation via BDNF/TrkB; Ca regulates synaptic enlargement/contraction (LTP/LTD). 2. Functional Improvement: Zn promotes neurogenesis; Ca upregulates synaptic genes via CaMKII/CREB; Cu/Zn improve NMDA receptor function.	Depression (reduced hippocampal BDNF, spine loss), Anxiety (aberrant amygdala plasticity), Schizophrenia (NMDA hypofunction)
**Gut–Brain axis regulation**	Zn, Mn	1. Zn's Role: Stabilizes gut barrier (tight junction proteins), modulates microbiota (increases beneficial bacteria), reduces LPS translocation and inflammation. 2. Mn's Role: Promotes intestinal *Lactobacillus* growth, enhances gut GABA synthesis, vagus nerve‐mediated central sedation.	Anxiety (dysbiosis, impaired barrier), Depression (gut‐origin inflammation), Neuropsychiatric Symptoms (insufficient gut GABA)

Abbreviations: BDNF, brain‐derived neurotrophic factor; Ca, calcium; CaMKII, Ca^2^^+^/calmodulin‐dependent protein kinase II; CREB, cAMP response element‐binding protein; Cu, copper; DA, dopamine; Fe, iron; GABA, gamma‐aminobutyric acid; HO‐1, heme oxygenase‐1; IL‐6, interleukin‐6; LPS, lipopolysaccharide; LTD, long‐term depression; LTP, long‐term potentiation; Mg, magnesium; Mn, manganese; NE, norepinephrine; NF‐κB, nuclear factor kappa‐light‐chain‐enhancer of activated B cells; NMDA, N‐methyl‐D‐aspartate; Nrf2, nuclear factor erythroid 2‐related factor 2; ROS, reactive oxygen species; TrkB, tropomyosin receptor kinase B; Zn, zinc.

The therapeutic action of Longgu is thus an emergent phenomenon. Ionostatic balance (Ca/Mg) provides fundamental stability, cross‐pathway synergy (e.g., Zn) coordinates functional correction with structural repair, and systemic integration (Zn/Mn) engages the gut–brain axis to resolve peripheral triggers and promote central calm. Through these collective, system‐level properties, this ancient mineral network achieves a therapeutic scope that modern single‐target pharmacology is yet to replicate.

## 4 Clinical Validation and the Network Pharmacology Perspective

The theoretical framework of the “Natural Synergistic Network” requires robust clinical validation. A growing body of evidence from studies and trials of Longgu‐containing formulations now provides compelling confirmation of this system‐level efficacy. This section synthesizes clinical data to demonstrate how the network's multi‐target actions translate into tangible patient benefits, particularly in complex cases refractory to conventional monotherapy. Examining these outcomes through a network pharmacology lens advances the inquiry from whether it works to how its synergistic mechanisms operate in a clinical context.

### 4.1 Clinical Efficacy: Evidence of Multi‐Domain Symptom Resolution

The most persuasive validation of the network hypothesis stems from the consistent observation that Longgu‐containing formulations produce improvements across a spectrum of co‐occurring symptoms, reflecting the multi‐domain action of the underlying ion consortium. Clinical profiles of key formulations, detailed in Table 5, demonstrate this broad‐spectrum efficacy.

**TABLE 5:** Observational clinical studies of representative longgu‐containing Chinese herbal compounds.

Formula name	Main components	Target condition	Study design (sample size/duration)	Core efficacy outcomes	Safety	References
Chaihu Jia Longgu Muli Tang	Bupleurum, Scutellaria, Pinellia, Ginger, Ginseng, Jujube, Cinnamon Twig, Poria, Rhubarb, Longgu, Oyster Shell	Insomnia with Depression, Anxiety	75 cases / Open Trial / 4 weeks	Reduction in HAMD and HAMA scores (decrease of 12.3 ± 2.8), superior to Zopiclone combined with Trazodone Hydrochloride tablets alone (*p* < 0.001)	No significant adverse reactions	[89]
Modified Chaihu Jia Longgu Muli Tang	Bupleurum, Longgu, Oyster Shell, Albizia Bark, Rose, Codonopsis, Poria, Cimicifuga, Acorus, Pinellia, Citrus Peel, Atractylodes, Polygala	Bipolar Disorder, Depressive Episode	76 cases / Open Trial / 6 weeks	Reduction in HAMD‐24 total score, TCM symptom scores (e.g., emotional depression), increased total sleep time; superior to SSRI alone	No significant adverse reactions	[90]
Modified Guizhi Longgu Muli Tang	Cinnamon Twig, Longgu, White Peony Root, Oyster Shell, Salvia, Jujube, Prepared Aconite, Pinellia, Ginger, Honey‐fried Licorice	Arrhythmia with Anxiety Disorder	74 cases / Open Trial / 12 weeks	Reduction in HAMA and PSQI scores; increased total effective rate	No significant adverse reactions	[94]
Modified Suanzaoren Tang and Guizhi Jia Longgu Muli Tang	Jujube Seed, Chuanxiong, Poria, Cinnamon Twig, Stir‐fried White Peony Root, Anemarrhena, Honey‐fried Licorice, Calcined Longgu, Calcined Oyster Shell	Insomnia with Anxiety	80 cases / Open Trial / 4 weeks	Reduction in Sleep Quality Index score and anxiety severity score	No significant adverse reactions	[117]
Modified Guizhi Gancao Longgu Muli Tang	Cinnamon Twig, White Peony Root, Honey‐fried Licorice, Longgu, Oyster Shell, Jujube, Salvia, Chuanxiong, Morinda, Epimedium, Alisma, Poria	Chronic Heart Failure with Sleep Disorders	60 cases / Open Trial / 4 weeks	Reduction in PSQI, MLHFQ scores; improvement in EF, CK‐MB, cTnT, BNP levels; increased 6‐minute walk test distance	No significant adverse reactions	[118]
Longgu‐containing multi‐herb formula	Longgu, Oyster Shell, Bupleurum, Scutellaria, Poria	Generalized anxiety disorder with insomnia	120 cases / Randomized controlled trial / 8 weeks	Significant reduction in HAMA scores (from 22.4 ± 3.1 to 9.8 ± 2.3, *p* < 0.01); improved PSQI scores; superior to buspirone control	Mild gastrointestinal discomfort in eight patients, resolved without intervention	[119]

Abbreviations: BNP, B‐type natriuretic peptide; CK‐MB, creatine kinase‐MB; cTnT, cardiac troponin T; EF, ejection fraction; HAMA, Hamilton Anxiety Rating Scale; HAMD, Hamilton Depression Rating Scale; MLHFQ, Minnesota Living with Heart Failure Questionnaire; PSQI, Pittsburgh Sleep Quality Index; SSRI, selective serotonin reuptake inhibitor; TCM, traditional Chinese medicine.

A prime example is Chaihu Jia Longgu Muli Tang for insomnia comorbid with depression and anxiety. In a clinical observation of 75 patients, this formula, combined with conventional treatment, yielded significantly greater reductions in both Hamilton Depression (HAMD) and Hamilton Anxiety (HAMA) scores. This was compared to a control receiving only zopiclone and trazodone [89]. This represents concurrent alleviation of both depressive and anxious symptomatology, not merely sedation. The proposed network mechanism involves the Ca/Mg duo stabilizing neuronal excitability, Zn balancing GABA/glutamate systems, and Fe/Cu supporting monoamine synthesis, thereby simultaneously addressing the pathologies of both conditions.

Similarly, a modified *Chaihu Jia Longgu Muli Tang* demonstrated superior outcomes to SSRI monotherapy in bipolar depressive episodes. Over 6 weeks, the group receiving the TCM formulation showed greater reduction in HAMD‐24 scores and TCM symptom scores, alongside increased total sleep time [90]. This profile of improved mood, sleep, and well‐being exemplifies multi‐target efficacy. While the SSRI provides a focused serotonergic boost, the Longgu network adds a crucial regulatory layer by potentially inhibiting HPA axis overactivity (via Mg), promoting neuroplasticity (via Zn/Ca), and dampening neuroinflammation (via Zn), resulting in a more comprehensive therapeutic response. Advances in neuroimaging have begun to characterize the neural circuit dysfunction underlying bipolar mood states, providing potential biomarkers for treatment response that may inform future studies of Longgu‐containing formulations in this population [91].

Efficacy extends beyond depression and anxiety. *Guizhi Gancao Longgu Muli Tang* and its modifications show significant benefits for palpitations and insomnia, improving sleep quality and reducing anxiety scores [92, 93, 94]. This can be interpreted as the network's ionostatic balance property stabilizing both myocardial and central neuronal excitability. Furthermore, formulations like *Longmu Zhuanggu Granules* increase peripheral Zn and Ca levels in children with anorexia nervosa, correlating with symptom improvement [83]. This provides direct biochemical evidence for the systemic absorption and activity of the network's components, contributing to restored physiological homeostasis.

A common theme across these studies is the particular effectiveness of Longgu formulations in managing comorbidity, such as depression with insomnia, anxiety with palpitations, or bipolar depression with sleep disturbances. This clinical scenario typically challenges single‐target drugs, often necessitating complex polypharmacy. The network's design inherently manages this complexity, as its components simultaneously engage the multiple dysfunctional systems underlying co‐occurring symptoms.

### 4.2 Biomarker Modulation: Tracing the Network's Footprint

Beyond symptom scales, clinical and preclinical studies demonstrate the network's direct impact on biomarkers, providing a physiological footprint of its multi‐target engagement. Research on *Chaihu Jia Longgu Muli Tang* shows its therapeutic effects coincide with measurable neurotransmitter changes. Clinical studies report the formulation significantly increases serum levels of the inhibitory neurotransmitter GABA and serotonin (5‐HT). It also reduces the excitatory neurotransmitter norepinephrine (NE) by up to 19.3% [92, 93]. This rebalancing of excitatory–inhibitory and monoamine tone is a direct clinical correlate of the combined actions of Zn (GABA enhancement, NMDA inhibition), Mg (NMDA blockade), and Fe/Cu (monoamine synthesis regulation).

Simultaneously, the network's impact on neuroinflammation is evident. In a rat epilepsy model, *Chaihu Jia Longgu Muli Tang* significantly reduced pro‐inflammatory cytokines IL‐6 and TNF‐α, an effect linked to downregulation of NF‐κB p65 and upregulation of its inhibitor, IκBa [95, 96]. This anti‐inflammatory action reflects Zn's known role in inhibiting the NF‐κB pathway and activating the Nrf2/HO‐1 axis [97, 98], demonstrating that the network concurrently resolves the inflammatory component of pathology alongside its effects on neurotransmission.

Evidence from *Longmu Zhuanggu Granules* further solidifies this link. The documented increase in peripheral Zn and Ca levels provides a direct pharmacokinetic connection between administration, systemic availability, and therapeutic effect [83]. Animal studies with this formulation demonstrate enhanced Ca absorption and transmembrane transport, alongside improved memory function, suggesting a direct pathway from ion supplementation to activation of Ca‐dependent plasticity mechanisms like the CaMKII/CREB pathway [80, 99]. Clinical biomarker studies have provided additional evidence for the multi‐target effects of Longgu‐containing formulations. A recent investigation demonstrated that treatment with Longgu‐based preparations was associated with significant changes in serum markers of oxidative stress, including reduced malondialdehyde and increased glutathione peroxidase activity, alongside improvements in mood and sleep parameters [88]. These findings suggest that the network's effects extend beyond neurotransmitter modulation to include redox regulation.

Collectively, this biomarker data presents a coherent picture: Longgu‐containing formulations induce a synchronized shift across multiple physiological domains, promoting a state of increased inhibitory tone, balanced monoamine activity, and reduced inflammation. This coordinated biomarker modulation is the definitive clinical signature of a multi‐target therapeutic agent.

### 4.3 The Network Pharmacology Perspective: Synergy With Conventional Treatment

The network pharmacology perspective provides a powerful framework for interpreting the enhanced efficacy of Longgu formulations alongside conventional Western medicines. The observed synergy with SSRIs is not merely additive but represents a potentiation arising from complementary mechanisms [90].

From a network pharmacology perspective, an SSRI is a high‐potency, specific intervention on a single pathological network node: the serotonin transporter (SERT). However, while powerful, this intervention fails to address other dysfunctional nodes. These include glutamate excitotoxicity, BDNF deficit, HPA axis overactivity, or neuroinflammation. Focusing exclusively on one node can even induce compensatory shifts that manifest as side effects or limited efficacy.

In contrast, the Longgu network is a broad‐spectrum, low‐intensity modulator of multiple nodes simultaneously. It does not block SERT with SSRI‐like potency, but it supplies Mg to dampen HPA axis and glutamate hyperactivity, Zn to boost BDNF and GABA, and other ions supporting metabolic and antioxidant functions. In combination, these approaches yield a superior outcome: the SSRI provides a strong, specific signal on the serotonergic node, while the Longgu network creates a permissive environment by stabilizing the entire system. It calms inflammatory noise, strengthens synaptic resilience, and stabilizes neuronal firing, thereby enabling more effective translation of the SSRI's signal into a healthy physiological state. Network analysis approaches have proven particularly valuable for understanding symptom interactions in psychiatric disorders. For instance, recent studies have mapped the interrelationships between depression, anxiety, and suicide ideation in adolescent populations, revealing central symptoms that may serve as efficient intervention targets [100, 101].

This explains the greater symptom reduction and improved tolerability often seen with combination therapy. The network mitigates side effects arising from a system struggling to adapt to a single, powerful perturbation. This synergistic model pairs a “targeted high‐potency agent” with a “system‐stabilizing network.” It represents a promising blueprint for future psychiatric pharmacotherapy. It suggests that optimal treatments for complex disorders may involve rational combinations of a targeted intervention and a systemic stabilizer, a strategy embodied by Longgu. Thus, clinical evidence not only validates the network hypothesis but also indicates its most impactful application in modern medicine. Table 5 provides an overview of observational clinical studies for representative Longgu‐containing formulations.

## 5 Translating the Blueprint: A Roadmap for Modern Therapeutics

The compelling clinical evidence and sophisticated network pharmacology of Longgu present a compelling proposition: the systematic reverse‐engineering of this ancient blueprint to create a new class of modern, evidence‐based psychiatric therapeutics. The translation from a complex mineral remedy to a standardized medicine presents significant scientific and regulatory challenges, which concurrently represent fertile ground for innovation. This section outlines a concrete translational roadmap, from fundamental research deciphering the network's principles to advanced engineering for targeted delivery and, ultimately, clinical validation. The objective is to transform Longgu from a TCM heritage into a pioneering model for 21st‐century network pharmacology.

### 5.1 Deciphering the Synergy Code: From Qualitative Description to Quantitative Prediction

The primary challenge in modernizing Longgu involves overcoming the uncertainties regarding its primary active ions, the principles governing their interactions, and their pharmacokinetic fate. Progress requires a shift from observational science to predictive, quantitative systems pharmacology. Central research questions include determining the optimal “efficacy ratio” of key ions for specific psychiatric indications. Additional questions involve characterizing their dynamic interactions in physiological environments. Finally, we must elucidate how organic components in complex formulations influence the bioavailability and targeting of the metal ion network.

A multi‐pronged research strategy is essential. First, advanced in vitro modeling using human induced pluripotent stem cell (iPSC)‐derived neuronal networks and brain organoids maintained in precisely controlled ion media is crucial. These systems are maintained in precisely controlled ion media. They enable the use of fractional factorial design and response surface methodology to systematically vary ion ratios (e.g., Ca, Mg, and Zn). Outputs such as neuronal firing patterns, synaptic density, BDNF release, and inflammatory cytokine production can then be quantified. This approach facilitates the mathematical modeling of interactions and identification of optimal synergistic ratios, building on foundational work demonstrating Zn and Mg synergy in neural stem cell biology [45, 75].

Second, integrated multi‐omics and metallomics represent a transformative approach. Metallomics is the comprehensive analysis of the metallome and its interactions with the genome, proteome, and metabolome. This field provides powerful tools for deciphering Longgu's complex mechanisms [102]. Combining ICP‐MS for precise ion quantification with transcriptomics, proteomics, and metabolomics allows for mapping the complete “component‐target‐pathway” network activated by the Longgu ion consortium. This integrated methodology can identify primary targets and, crucially, the downstream cascades uniquely engaged by the ion combination versus individual elements. Thereby, it reveals the systems‐level effects underlying the network's therapeutic efficacy. Network pharmacology approaches have proven valuable for analyzing complex natural product combinations. A recent study applying systems biology methods to traditional mineral‐containing formulations demonstrated that therapeutic effects emerge from coordinated interactions among multiple constituents rather than from individual components acting in isolation [103]. This analytical framework is directly applicable to understanding how Longgu's metal ion network produces its multi‐target effects.

Third, comprehensive pharmacokinetic mapping using isotopic tracers is essential to characterize the poorly understood in vivo disposition of Longgu's ions. Incorporating stable or radioactive isotopes into processed Longgu or its extracts enables precise tracking of absorption, distribution across the blood‐brain barrier, accumulation in specific brain regions, and excretion [104, 105]. Coupled with spatial analysis techniques like laser ablation–ICP‐MS (LA–ICP‐MS), this approach can generate detailed, spatially resolved maps of ion distribution in brain tissue, revealing the primary neural circuits targeted by the network [102]. This knowledge is fundamental for optimizing delivery strategies and understanding the spatial dynamics of the network's therapeutic effects. Key applicable metallomics techniques are outlined in Table 6.

**TABLE 6:** Applications of metallomics analysis techniques [102].

Analytical technique	Core technical characteristics	Application value in longgu research
Inductively Coupled Plasma Mass Spectrometry (ICP‐MS)	Extremely high sensitivity (detection limits in ppb–ppt range), supports simultaneous multi‐element quantification, can provide isotopic information.	Precisely determines the content of over 20 metal ions in Longgu, clarifying the total elemental characteristics.
Neutron Activation Analysis (NAA)	Non‐destructive detection, requires minimal sample preparation, no matrix interference, low detection limits (10−^6^‐10−^9^ g/kg).	Suitable for in‐situ quantitative analysis of metal elements in natural mineral drugs like Longgu, avoiding element loss or contamination from sample pretreatment.
Laser Ablation–ICP‐MS (LA–ICP‐MS)	Can directly analyze solid samples, strong micro‐area analysis capability, spatial resolution up to ∼100 µm, provides spatial distribution information for elements.	Clearly visualizes distribution differences of metal ions in different internal regions of Longgu, revealing microscopic occurrence patterns of elements.
X‐ray Fluorescence Spectroscopy (XRF)	Non‐destructive multi‐element analysis, micro‐beam x‐ray enables micron‐level spatial resolution imaging, requires minimal sample preparation.	Rapidly detects metal ion distribution on the surface and cross‐section of Longgu, efficiently obtaining macro‐ and micro‐distribution characteristics of elements.
Particle‐Induced x‐ray Emission (PIXE)	Capable of multi‐element detection, micron‐level spatial resolution, can directly analyze solid samples, simple sample pretreatment.	Suitable for micro‐area metal distribution detection in mineral drugs like Longgu, intuitively presenting local enrichment or depletion states of elements within the sample.

### 5.2 Targeted Delivery of the Network: Nanotechnology as a Key Enabler

A critical translational hurdle is the poor bioavailability and lack of brain‐specific targeting of orally administered metal ions. While delivering a single ion to the brain is challenging, delivering a complex, synergistic ratio constitutes a formidable task requiring a paradigm shift in formulation science. Nanotechnology offers powerful tools to overcome these delivery challenges via two primary strategic approaches.

The first strategy involves sophisticated nanocarrier construction and targeted modification. Biodegradable, biocompatible polymeric nanoparticles, particularly those made from poly(lactic‐co‐glycolic acid) (PLGA), are ideal candidates for co‐encapsulating multiple metal ions in their identified synergistic ratios [106]. A core innovation challenge is developing fabrication methods that ensure consistent and precise loading of this “efficacy ratio.” Furthermore, surface functionalization of these nanocarriers with brain‐targeting ligands enables active targeting that significantly enhances crossing efficiency. Examples of such ligands include TAT or Angiopep‐2 peptides, as well as antibodies against BBB receptors like the transferrin receptor [106]. This strategy concentrates the ion network at its site of action while minimizing systemic off‐target effects and potential toxicity, thereby maximizing the therapeutic index.

The second strategy employs intelligent responsive release systems designed to mimic the context‐dependent ion release of Longgu's natural matrix. These “smart” nanocarriers can be engineered to release their payload in response to specific pathological stimuli [107, 108]. pH‐responsive systems maintain stability at physiological pH but disassemble in the acidic microenvironment of the gastrointestinal tract or inflamed tissues, enhancing site‐specific absorption. Reactive oxygen species (ROS)‐responsive systems, constructed with thioketal or similar linkers, degrade and release antioxidant ions precisely in brain regions with high ROS levels, providing targeted anti‐inflammatory and neuroprotective effects. Enzyme‐responsive systems, designed for cleavage by enzymes overexpressed in disease states (e.g., matrix metalloproteinases in areas of synaptic remodeling), offer another layer of specificity, ensuring the network's components are activated in the appropriate pathological context. Recent advances in metal–organic frameworks (MOFs) and coordination chemistry have enabled precise control over metal ion release profiles. Studies demonstrate that calcium and zinc complexes can be engineered to provide sustained delivery while maintaining bioavailability [109, 110, 111]. These approaches may be adapted to replicate Longgu's synergistic ion ratios in a precisely controlled pharmaceutical formulation.

MOFs represent an innovative approach for delivering therapeutic metal ions with precise spatial and temporal control. Recent developments in the synthesis of zinc‐based MOFs demonstrate their potential for controlled release in biological environments, with degradation rates tunable through framework composition and structure [110]. Such platforms could potentially be engineered to deliver the specific ion ratios identified as optimal in Longgu research.

### 5.3 Quality Standardization and Clinical Translation: Bridging the Gap to the Clinic

For Longgu to achieve acceptance into modern pharmacopeias, it must overcome issues of variable composition and the absence of efficacy‐based quality control. Establishing a new quality control paradigm is foundational for successful clinical translation. This requires a comprehensive approach to standardization and safety.

Current standards in the Chinese Pharmacopoeia are insufficient, focusing on macroelements while lacking specifications for the neuroactive trace elements critical to Longgu's therapeutic properties  [112]. A modern quality control system must be multi‐dimensional and scientifically rigorous. It should define permissible ratio ranges for key ion pairs based on demonstrated synergistic efficacy, thereby embracing the network pharmacology principles underlying Longgu's action. Implementing bioactivity‐based assays represents another crucial advancement, shifting quality control from chemical quantification to functional assessment of therapeutic potential. For example, a Longgu extract's capacity to induce BDNF expression in neuronal cultures or inhibit LPS‐induced NF‐κB activation in microglia could serve as batch‐to‐batch efficacy benchmarks, ensuring consistent pharmacological activity. Furthermore, given its fossil origin, establishing and strictly enforcing safety limits for geogenic contaminants like lead (Pb), cadmium (Cd), and arsenic (As) is non‐negotiable for patient safety and regulatory compliance [23, 24].

The clinical translation can proceed through three strategic pathways. The first involves developing standardized single‐ingredient preparations by formulating purified Longgu extracts into modern dosage forms like sustained‐release tablets or capsules. These preparations are suitable for initial regulatory approval and for treating mild‐to‐moderate anxiety and insomnia. They provide a well‐characterized therapeutic that embodies the network principle.

The second pathway focuses on quality‐standardized compound formulations that preserve traditional wisdom while ensuring modern quality standards. For classic prescriptions like Chaihu Jia Longgu Muli Tang, this employs advanced analytical techniques, including HPLC fingerprinting combined with ICP‐MS, to establish comprehensive chemical and elemental fingerprints for quality consistency [113]. Critically, this pathway requires defining permissible ratio ranges of key metal ions to organic markers to ensure consistent batch‐to‐batch quality and reproducible therapeutic effects, maintaining the synergistic network's integrity within modern pharmaceutical standards.

The third, and perhaps most immediately promising, pathway involves rational combination therapies, systematically investigating Longgu as an adjunct to first‐line Western medicines. Building on clinical evidence of enhanced efficacy when combined with SSRIs [90], this approach warrants large‐scale, randomized controlled trials to validate and quantify the synergy. This strategy leverages complementary strengths: the high potency and specificity of targeted Western drugs combined with the system‐stabilizing, multi‐target capacity of the natural ion network, potentially offering superior outcomes for complex or treatment‐resistant conditions. An integrated summary of evidence and the proposed research roadmap are consolidated in Table 7.

**TABLE 7:** Integrated evidence and future research roadmap for longgu.

Evidence summary for longgu‐containing formulations		
Formulation	Key findings (related to metal ions)	Proposed mechanism linked to metal ions
Chaihu Jia Longgu Muli Tang (Clinical: Depression)	Increased serum GABA, 5‐HT; reduced NE [92, 93].	Ca/Mg/Zn regulation of neurotransmitter release and receptor balance.
Chaihu Jia Longgu Muli Tang (Preclinical: Epilepsy)	Reduced IL‐6, TNF‐α; modulated NF‐κB pathway [95, 96]	Zn‐mediated anti‐inflammatory effects via Nrf2/HO‐1 and NF‐κB inhibition.
Guizhi Gancao Longgu Muli Tang (Clinical: Anxiety, Insomnia)	Improved sleep quality, reduced anxiety scores [92, 93].	Ca/Mg‐mediated stabilization of neuronal excitability; Zn enhancement of GABAergic transmission.
Longmu Zhuanggu Granules (Clinical/Preclinical)	Increased peripheral Zn, Ca levels; improved memory [83, 99].	Direct supplementation; Zn via BDNF/TrkB; Ca via CaMKII/CREB for synaptic plasticity.
Longgu + SSRI (Combination Therapy) (Clinical: Depression)	Greater reduction in HAMD‐24 scores vs. SSRI alone [90].	Multi‐target synergy: SSRI targets SERT, while Longgu ions address inflammation, neuroplasticity, and complementary neurotransmitter systems.
**Future research roadmap**		
**Research priority**	**Key questions**	**Recommended approaches/technologies**
1. Deciphering synergy	What is the optimal “efficacy ratio” of Ca:Mg:Zn? How do ions interact with organic TCM components?	‐ In vitro neuronal cultures with controlled ion media. ‐ Fractional factorial design. ‐ Metabolomics. ‐ Network pharmacology analysis [103]
2. Mapping in vivo disposition	How are Longgu's metal ions absorbed, distributed, and accumulated in the brain?	‐ Radioisotope tracing (e.g., ^4^ ^5^Ca, ^6^ ^7^Zn). ‐ LA‐ICP‐MS imaging of brain tissue. ‐ Gut microbiome metagenomics.
3. Target engagement & validation	Which specific neural targets (VGCC subtypes, NMDA subunits, Nrf2) are most critical?	‐ CRISPR‐based gene editing. ‐ Phosphoproteomics. ‐ Surface Plasmon Resonance (SPR).
4. Advanced Formulation	Can we design a nano‐formulation that mimics Longgu's natural ion ratio and targets the brain?	‐ PLGA/Mesoporous Silica nanoparticles. ‐ Metal–organic frameworks [110] ‐ Controlled‐release metal complexes [111] ‐ Functionalization with BBB‐targeting peptides (e.g., TAT). ‐ In vivo efficacy testing.
5. Future therapeutic directions	How can Longgu inform next‐generation psychiatric treatments?	‐ Network‐derived multi‐target therapeutic design [114]

Looking toward future therapeutic applications, emerging concepts in network pharmacology suggest that optimal treatments for complex psychiatric disorders may involve rationally designed combinations of targeted agents and system‐stabilizing interventions. A forward‐looking perspective on psychiatric drug development emphasizes the potential of multi‐target approaches derived from natural product networks [114]. Longgu embodies this principle and may serve as a template for developing next‐generation therapeutics that address the multifactorial nature of mental illness.

Overall, translating the Longgu blueprint is a multidisciplinary endeavor integrating metallomics, nanotechnology, systems biology, and clinical psychiatry. The roadmap is delineated: first, decipher the synergy code using advanced in vitro and omics technologies; second, engineer targeted delivery systems via smart nanotechnology to overcome bioavailability hurdles; and third, establish robust quality standards and clinical validation protocols. By following this path, the ancient wisdom of Longgu can be realized as a foundational inspiration for next‐generation, multi‐target psychiatric therapeutics that address mental disorder complexity at a systems level.

## 6 Conclusion and Future Perspective

This review has evaluated the hypothesis that Longgu's therapeutic effects in psychiatric disorders arise from synergistic interactions among its constituent metal ions. The evidence suggests that calcium, magnesium, zinc, iron, copper, and manganese function collectively to stabilize neuronal excitability, support neuroplasticity, and modulate gut–brain communication. Clinical studies of Longgu‐containing formulations demonstrate improvements across multiple symptom domains, particularly in patients with comorbid conditions. These studies also indicate potential synergy with conventional antidepressants.

Several research priorities emerge from this analysis. First, quantitative studies are needed to establish optimal ratios of key ions for specific therapeutic applications. Second, the pharmacokinetics and brain distribution of Longgu‐derived ions require characterization using isotopic tracing and spatial imaging techniques. Third, quality control standards should be expanded to include trace element profiles and bioactivity‐based assays. Fourth, nanotechnology‐based delivery systems warrant investigation as a means to improve ion bioavailability and brain targeting.

The clinical development pathway could proceed through standardized Longgu preparations for mild‐to‐moderate symptoms, quality‐controlled compound formulations preserving traditional combinations, and rigorous trials of Longgu as adjunctive therapy with conventional psychotropics. While significant scientific and regulatory challenges remain, the conceptual framework of multi‐target network therapy derived from Longgu offers a potentially valuable approach for addressing the complex pathophysiology of psychiatric disorders.

## Authors Contributions

**Sun Xiujia** and **Li Zhan Hua**: writing – original draft, writing – review and editing, data curation, formal analysis, resources, investigation, and methodology. **Gao Yuanze**: data curation, formal analysis, resources. **Liu Jing**: data curation, formal analysis, resources. **Li Li**: resources and data curation. **Zhang Chao, Kumar Ganesan, and Jianping Chen**: conceptualization, writing – original draft, writing – review and editing, methodology, project administration, supervision, resources, and data curation.

## Funding

The authors have no funding to disclose.

## Conflicts of Interest

The authors declare no conflicts of interest.

## Use of Generative AI and AI‐Assisted Technologies in the Writing Process

During the preparation of this manuscript, the authors used ChatGPT (OpenAI, GPT‐4o) for the purposes of language refinement, structural suggestions, and generating draft summaries for tables and figures. All AI‐generated content was thoroughly reviewed, edited, and validated by the authors, who take full responsibility for the final content of this publication.

## Data Availability

Data sharing not applicable to this article as no datasets were generated or analyzed during the current study.
